# Evolution of artificial intelligence and machine learning in DILI toxicogenomics: from descriptive profiling to mechanistic insights

**DOI:** 10.3389/fphar.2026.1907884

**Published:** 2026-08-12

**Authors:** Mohammad Sujaur Rahman, Minjun Chen

**Affiliations:** 1 Department of Information Science, University of Arkansas at Little Rock, Little Rock, AR, United States; 2 Division of Bioinformatics and Biostatistics, US FDA’s National Center for Toxicological Research, Jefferson, AR, United States

**Keywords:** artificial intelligence, drug-induced liver injury, generative AI, hepatotoxicity, machine learning, mechanism-informed prediction, toxicogenomics, transcriptomics

## Abstract

Drug-induced liver injury (DILI) is a critical safety issue in drug development, characterized by its idiosyncratic nature, complex mechanisms, and poor predictability in standard preclinical models. High-dimensional omics strategies, particularly toxicogenomics, have attracted increased interest in addressing the complexity of hepatotoxicity, especially in the context of emerging artificial intelligence (AI) technologies. This review traces the evolution of AI and machine learning (ML) within DILI-related omics research, highlighting toxicogenomics as a primary driver of advancement in this field. We first explore the early studies in computational toxicogenomics, which primarily focused on exploratory approaches, utilizing clustering, time-series, co-expression, and basic pathway analyses to identify molecular signatures indicative of nascent liver injury. We then examine how the adoption of supervised machine learning enabled robust predictive modeling, facilitating systematic feature selection, signature refinement, and rigorous validation. More recently, the field has been further transformed by deep learning, biologically informed network architectures, and generative artificial intelligence. Across these methodological eras, AI has enhanced mechanistic interpretation by identifying biologically relevant signatures, integrating multimodal evidence, and strengthening evidence for established DILI mechanisms, including oxidative stress, mitochondrial dysfunction, altered xenobiotic metabolism, inflammation, and cell death. Ultimately, the synergy of AI and DILI toxicogenomics has transitioned the discipline from descriptive profiling toward mechanism-driven predictive toxicology.

## 1 Introduction

DILI remains a major challenge in drug development and clinical practice (Chalasani et al., 2025; Andrade et al., 2019; Chen et al., 2014). DILI is one of the leading causes of drug development failures, restricted usage, and market withdrawals (Moreno-Torres et al., 2024; Chen et al., 2011). Preclinical studies are particularly difficult for predicting idiosyncratic DILI, which has highly variable susceptibility in patients and the underlying mechanisms are not fully understood (Chierici et al., 2020; Liu et al., 2020). The spectrum of DILI extends from abnormalities in liver function tests to severe hepatitis, acute liver failure and death (Suzuki and Chen, 2025). Further, the common clinical classification of DILI based on transaminitis does not adequately capture mixed phenotypes or progression over time or variability between individuals. There are therefore substantial gaps in knowledge that need to be addressed regarding accurate prediction of hepatotoxicity (Shrimali et al., 2025; Quintás et al., 2021).

The integration of high-dimensional omics strategies—particularly transcriptomics—has become increasingly pivotal in deciphering the multifaceted nature of DILI. This paradigm shift was catalyzed by the foundational premise that molecular alterations often precede observable phenotypic changes. Afshari et al. (1999) first proposed that shifts in gene expression patterns occur well before the onset of clinical pathological symptoms. This was further substantiated by Roth et al. (2011), who demonstrated significant transcriptomic perturbations in rat livers treated with a histamine-3 receptor antagonist, even in the absence of histopathological evidence of toxicity. These insights fostered the expectation that toxicogenomics could identify “show-stopping” toxicities, such as DILI, during the early stages of drug discovery (Ruepp et al., 2005), thereby allowing for the attrition of candidates with unacceptable safety margins (Yang et al., 2004). The progress of the field was significantly accelerated by the public release of two massive toxicogenomics repositories: the Japanese Toxicogenomics Project (TG-GATEs) (Uehara et al., 2010) and DrugMatrix (Ganter et al., 2005). These databases, as detailed by Chen et al. (2012), provided the critical mass of data necessary to transition the field from descriptive studies toward predictive and mechanistic toxicology.

Toxicogenomics also allows pathway-level and mechanistically informative analysis beyond conventional single-endpoint *in vitro* toxicity assays (De Abrew et al., 2015; Liu et al., 2015; García- Cañaveras et al., 2016; Bergen et al., 2025; Hellgren et al., 2026). It has been further shown by toxicogenomic studies that transcriptomic changes associated with liver injury could be detected outside the liver tissue but rather in accessible matrices like blood (Zhang L. et al., 2012). The use of transcriptomics has also supported mode-of-action analysis, pathway-based interpretation, and comparison of responses across biological systems (De Abrew et al., 2015; Sutherland et al., 2016). The expansion of omics, however, created new analytical challenges because toxicogenomic datasets are high-dimensional and not easily reducible to interpretable biological signals by conventional means. In particular, very large feature sets result from transcriptomic studies, which pose practical implementation and high-throughput testing challenges (Rueda-Zárate et al., 2017; Jiang et al., 2023). This has led to AI and ML being increasingly applied in toxicogenomics especially for tasks such as classification, prediction, feature selection, and biomarker discovery.

Following an outline of our literature selection framework (Section 2), this review traces the computational evolution of toxicogenomics from early pattern discovery to modern generative AI (Section 3). We then synthesize the key mechanistic insights these models have yielded regarding DILI (Section 4), before concluding with a critical analysis of current translational bottlenecks (Section 5) and future directions (Section 6).

## 2 Scope, literature selection, and review framework

**FIGURE 1:**
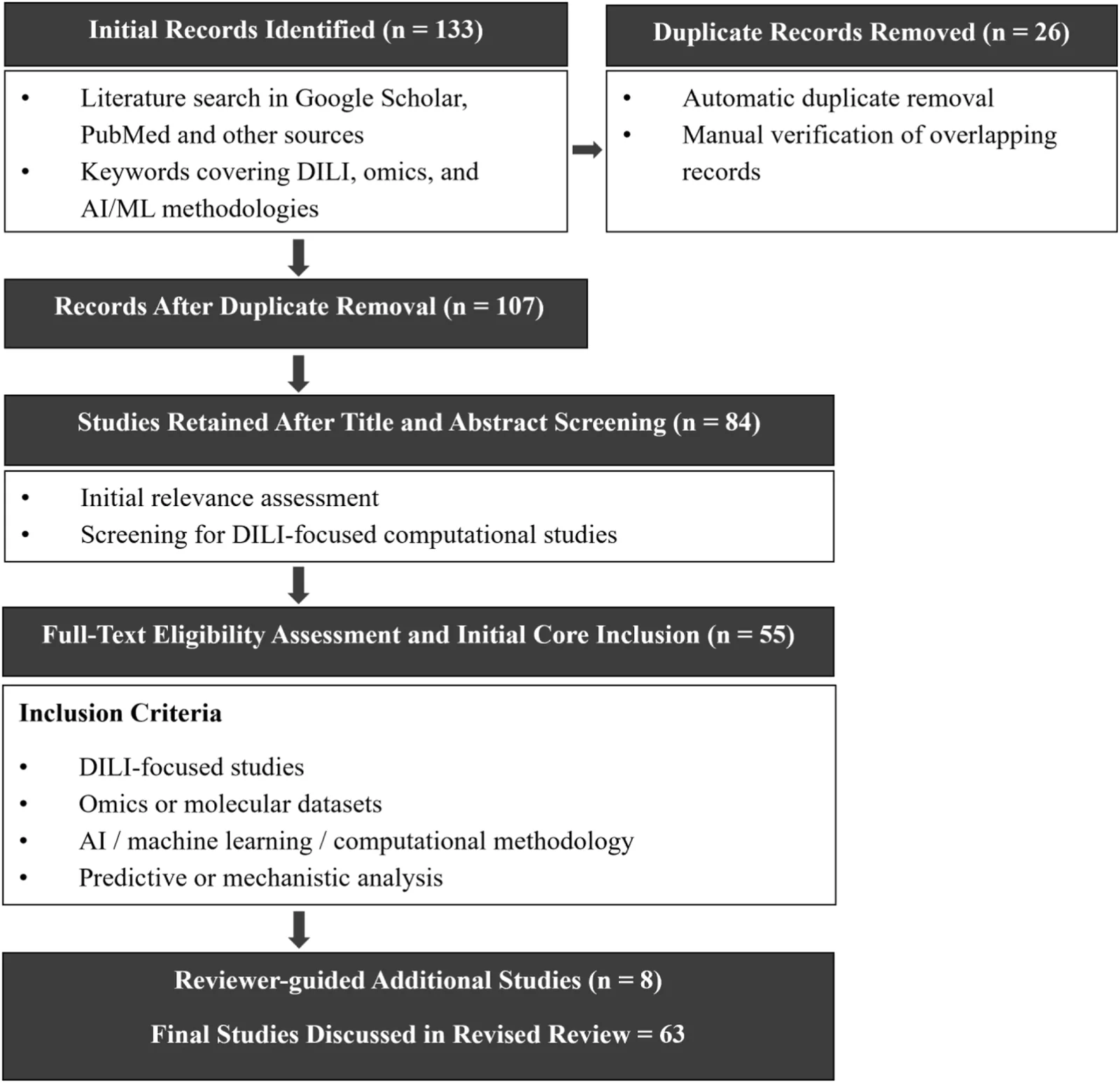
Workflow for literature identification, screening and final study selection.

We conducted a comprehensive literature search across Google Scholar and PubMed using specific keyword combinations, including DILI, hepatotoxicity, transcriptomics, toxicogenomics, omics, machine learning, artificial intelligence, deep learning, network analysis, graph theory, and multimodal modeling. This initial search yielded 133 papers, which were subsequently refined to 107 unique publications. Titles and abstracts were used to screen the papers more closely. Papers that applied computational approaches using ML or AI techniques on molecular data pertinent to DILI were prioritized. Studies were included if they met the following criteria: (i) the study focused on DILI, hepatotoxicity, or liver injury-relevant toxicity endpoints; (ii) the analysis used omics or toxicogenomic data, particularly transcriptomic, pathway-level, network-based, or multimodal toxicology data; (iii) the study applied AI, ML, deep learning, network modeling, or related computational methods; and (iv) the work contributed to prediction, biomarker discovery, mechanistic interpretation, or translational modeling. Studies were excluded if they were based only on clinical chemistry, chemical structure, or conventional toxicology endpoints without omics integration, or if they did not apply an AI/ML or computational modeling framework. A total of 55 papers were selected during the initial screening and formed the core basis for this review. During revision, eight additional targeted papers were incorporated in response to reviewer comments to improve coverage of multimodal integration beyond transcriptomics, proteomics, explainable AI, graph-based interpretability, non-GAN generative architectures, and clinical/translational context. Therefore, 63 papers were finally discussed in the revised review (Figure 1).

Although toxicogenomics can broadly include multiple omics layers, this review focuses primarily on transcriptomic and gene-expression-based toxicogenomic studies because most AI/ML frameworks for DILI-related toxicogenomics have been developed using transcriptomic perturbation datasets, including TG-GATEs, DrugMatrix, LINCS L1000, CMap, and related public resources. Metabolomics, proteomics, microbiome, and other omics studies were considered only when they were directly integrated with AI/ML-based DILI prediction, mechanistic interpretation, or multimodal toxicogenomic modeling. Studies based primarily on metabolomics or proteomics without a transcriptomic component, without an AI/ML modeling framework, or without a DILI-relevant toxicogenomic endpoint were excluded from the main methodological synthesis. This focus was chosen to maintain a coherent review scope and to allow comparison across studies using broadly similar molecular data types, while acknowledging that other omics layers represent important complementary directions for future DILI modeling.

Single-cell transcriptomic studies were not included as a dedicated category because the present review focused primarily on AI/ML applications in bulk toxicogenomic, perturbational transcriptomic, and multimodal toxicology datasets. However, we acknowledge that the absence of single-cell transcriptomics represents an important scope limitation.

## 3 Evolution of AI and ML in DILI toxicogenomics

**FIGURE 2:**
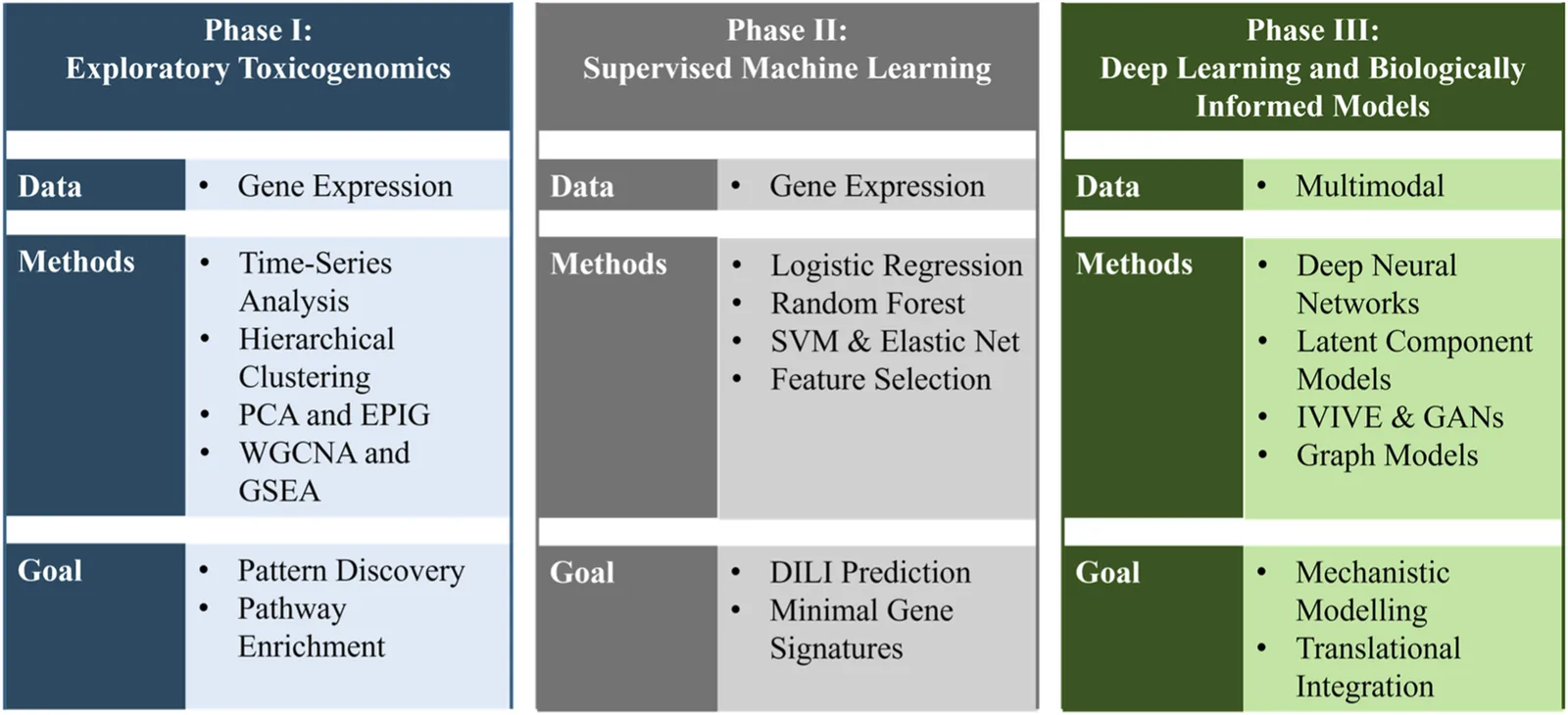
Evolution of AI and ML approaches in DILI toxicogenomics across three methodological phases. The blue column represents exploratory toxicogenomics, highlighting the types of transcriptomic data analyzed, the computational methods employed, and their primary analytical goals. The gray column represents supervised machine learning, emphasizing predictive modeling, feature selection, and biomarker discovery. The green column represents deep learning and biologically informed models, including multimodal learning, network-based approaches, and mechanism-oriented prediction. Abbreviations: PCA, principal component analysis; EPIG, extracting patterns and identifying co-expressed genes; WGCNA, weighted gene co-expression network analysis; GSEA, gene set enrichment analysis; SVM, support vector machine; GANs, generative adversarial networks; IVIVE, *in vitro* to *in vivo* extrapolation.

AI and ML applications in modern DILI toxicogenomics have accelerated over the past decade. The initial studies were mainly concerned with discovering biochemical patterns in transcriptomic data. This was followed by ML for prediction and biomarker identification. More recently, studies have advanced toward deep learning as well as biologically informed models that integrate pathways, networks, and multimodal data (Figure 2). Representative studies, data sources, validation strategies, and reported performance metrics are summarized in Table 1. However, these phases should be interpreted as overlapping methodological eras rather than strictly sequential stages. Earlier approaches such as clustering, pathway enrichment, and co-expression analysis continue to be used in recent studies, often as interpretability layers within supervised or deep learning workflows. Similarly, some studies span more than one phase; for example, probabilistic component modeling and biologically informed feature spaces can combine predictive modeling with representation learning. Thus, the framework is intended as a heuristic for organizing methodological evolution rather than a rigid chronology.

**TABLE 1:** Summary of representative studies included in the methodological evolution of AI/ML in DILI transcriptomics and toxicogenomics.

Study	Data source	Sample size	AI/ML methodologies	Validation strategy	Performance metrics and results
De Abrew et al. (2015)	Primary rat hepatocytes; TG-GATEs comparison	10 hepatotoxic compounds; TG-GATEs validation with 170 compounds	Hierarchical clustering; gene Ontology gene-set enrichment; pathway-filtered reclustering	External validation with TG-GATEs	Grouped compounds by toxicological MOA with good in vitro–in vivo concordance
Sutherland et al. (2016)	DrugMatrix, open TG-GATEs, and GEO	Rat liver, rat/human primary hepatocytes, mouse liver, HepG2 datasets	Gene-level analysis; gene set analysis (GSA); weighted gene co-expression network analysis (WGCNA)	Cross-dataset and cross-species concordance analysis	WGCNA improved cross-system concordance compared with gene- or pathway-level analyses
Chierici et al. (2020)	CMap/CAMDA 2018; MCF7 and PC3	266 compounds; 187 training, 79 external test	Deep learning; random forest; multilayer perceptron	5-fold CV plus external validation	Poor external performance, validation MCC generally <0.2
Liu et al. (2020)	DrugDex, DrugPoints/DailyMed labels; PubChem/DrugBank/ATC; LINCS L1000	1,317 DrugDex, 372 DrugPoints, 902 DailyMed drugs	Logistic regression; random forest; feature comparisons	10-fold CV plus 20% independent test set	Best models achieved AUC around 0.8
Aguayo-Orozco et al. (2021)	CMap/CAMDA 2018; MCF7 and PC3	276 compounds; 190 training, 86 blind validation	SVM; random forest	Repeated 10-fold CV plus blind validation	Pathway-based SVM performed best; validation accuracy 0.95 in MCF7 and 0.90 in PC3; MCC = 0.74
Jiang et al. (2023)	Open TG-GATEs primary human hepatocytes (PHH) (train) + GEO GSE28878 (HepG2 external)	18 training compounds; 10 external validation compounds	Multi-model ML; wrapper feature selection sequential backward selection/sequential forward selection/recursive feature elimination; linear regression	CV plus external validation	13-Gene DIC signature achieved external accuracy around 0.71
Zhao et al. (2024)	Open TG-GATEs rat liver, CMap L1000 PHH for screening	123 TG-GATEs compounds; 185 CMap compounds for screening	Elastic net logistic regression; random forest; signature scoring	Hold-out test set plus independent CMap screening	High-dose model achieved AUC ≈0.71 and ∼71% hold-out accuracy
Kohonen et al. (2017)	CMap + NCI-60 + TG-GATEs + DILI-annotated drugs	1,300 compounds; 68 DILI-annotated drugs; 1,331 PTGS genes	Probabilistic component modeling; semi-supervised PTGS	External validation with cytotoxicity assays, rat liver pathology, and human DILI annotation	PTGS predicted cytotoxicity, rat liver pathology, and human DILI; DILI sensitivity ∼72–86%
Li et al. (2020)	LINCS L1000 + DILIst	6,000 profiles 4,800 training, 1,200 independent validation	DNN; Monte Carlo cross-validation (MCCV); permutation test; pathway analysis	100-Iteration MCCV, permutation test, independent validation	DNN performed best; AUC = 0.798, balanced accuracy = 0.721, sensitivity = 0.839, specificity = 0.603
Jaume et al. (2024b)	TG-GATEs rat liver: whole-slide histology + microarray	10,234 image-expression pairs; ∼8,232 training slides, 2,002 held-out test slides	Multiple instance learning; gene expression imputation; lesion classifier	Held-out test	Linked six liver lesions to gene signatures; lesion classifier achieved AUC of 98.9%

Abbreviations: CV, cross-validation; DILI, drug-induced liver injury; ML, machine learning; AUC, area under curve; MCC, matthews correlation coefficient; SVM, support vector machine.

### 3.1 Early toxicogenomic studies for pattern discovery

In the early phase of DILI toxicogenomics, the domain mainly focused on transcriptomic pattern discovery, aiming to identify gene expression patterns in dose-response experiments associated with hepatotoxic compound treatments, phenotypes, or physiological states. The primary computational techniques included time series ranking, hierarchical clustering, principal component analysis, gene set enrichment analysis, and co-expression module analysis. For example, Rueda-Zárate et al. (2017) analyzed TG-GATEs microarray data from rat *in vivo*, rat *in vitro*, and human *in vitro* systems. Their workflow integrated time-series analysis with multivariate empirical Bayes ranking followed by unsupervised hierarchical clustering and pre-ranked gene set enrichment analysis to classify hepatotoxic compounds based on patterns of gene-expression.

De Abrew et al. (2015) assessed if primary rat hepatocytes could be used as an *in vitro* transcriptomic model for predicting *in vivo* hepatotoxic mode of action. Their analytical workflow included differential-expression analysis, hierarchical clustering, gene-set enrichment based on Gene Ontology (GO) annotations, and a final pathway-informed reclustering step. Using the 24-h high-dose condition selected for downstream analysis, this study found biologically meaningful groupings such as peroxisome proliferators and cytochrome P450 inducers. This is an early example of pathway-informed transcriptomic grouping in hepatotoxicity assessment.

Tawa et al. (2014) identified co-expressed gene modules related to liver injury endpoints using DrugMatrix database. Seventy-eight modules were generated with the iterative signature algorithm that were related to indicators of injury and chemical classes, which were used for defining the role of gene sets in liver injury as well as developing new signatures for fibrosis, steatosis, and general injury. These modules were externally validated in TG-GATEs and datasets from Gene Expression Omnibus (GEO). Zhang L. et al. (2012) aimed to assess whether whole-blood toxicogenomics could serve as an alternative surrogate approach for acetaminophen hepatotoxicity. Using the “extracting patterns and identifying co-expressed genes” (EPIG) on rat liver and blood, they highlighted co-regulated gene patterns in common that are associated with two sample types. Early gene signatures were able to distinguish between subtoxic and toxic exposures, while later gene signatures were related to biological injury processes in liver.

Although these studies are predominantly proof-of-concept in nature, they established a basic framework by demonstrating that liver toxicity possesses a recoverable transcriptomic structure.

### 3.2 Machine learning for predictive toxicity and biomarker identification

DILI toxicogenomic studies moved toward prediction and biomarker discovery to determine whether biological patterns could be leveraged for accurately predicting DILI risk at the early stage (Zhang M. et al., 2012; Liu et al., 2019). Logistic regression (LR), random forest (RF), support vector machine (SVM), and gradient boosting machine (GBM) were the most used ML algorithms.

Liu et al. (2020) utilized various measurements for DILI prediction, such as chemical descriptors, Anatomical Therapeutic Chemical (ATC) codes, binding proteins, and Library of Integrated Network-Based Cellular Signatures (LINCS) L1000 gene-expression features. The analysis revealed that ATC codes and protein binding features were more predictive than gene-expression features. Among DILI endpoints, severe cases such as fatal hepatic adverse drug reactions and liver failure were found to have higher predictability compared to others. Stringently defined negative samples turned out to be critical for predictability.

Other transcriptomics studies aimed at improving prediction with biologically relevant inputs. Aguayo-Orozco et al. (2021) used SVM and RF algorithms to develop QGexAR models on gene-expression data from MCF7 and PC3 cell lines. Pathway-level Kyoto Encyclopedia of Genes and Genomes (KEGG) signatures were found to be the best in external validation after filtering thousands of genes down to just 171. This study shows that the predictive performance of supervised ML methods benefits from biologically filtering transcriptomics inputs. Aguirre-Plans et al. (2021) applied supervised ML in the Critical Assessment of Massive Data Analysis (CAMDA) 2019 competition and found that raw Connectivity Map (CMap) transcriptomics features performed poorly, while phenotype-guided features and combinations with chemical structure improved robustness.

Some studies focused on identifying minimal gene signatures for DILI relevant phenotypes. Smith et al. (2020) utilized supervised ML approaches to develop feature selection and predictive models based on the rat liver TG-GATEs dataset and testing. They identified a set of ten gene biomarkers that could accurately predict the presence of liver necrosis. Jiang et al. (2023) investigated drug-induced intrahepatic cholestasis using Open TG-GATEs and external validation through GEO. Logistic regression with sequential backward selection outperformed eight other classifiers and provided a minimal 13-gene signature that had an average prediction accuracy of 0.71 in external validation.

Zhao et al. (2024) proposed INSIGHT, an elastic-net-based framework that used 24-h rat liver transcriptomic profiles from Open TG-GATEs to define a 235-gene DILI-related signature for predicting drug-induced hepatotoxicity. Shin et al. (2025) showed that gene expression alone had limited capability for predicting DILI risk, but targeted DILI-related gene sets or transcriptomic models combined with chemical structure data improved predictive performance.

Overall, these studies suggest that biologically structured inputs, such as pathway-level features, phenotype-guided factors, and selected gene signatures, perform better than raw gene-expression matrices alone. This likely reflects reduced noise, lower dimensionality, and better preservation of coordinated hepatotoxicity-related biology.

### 3.3 Deep learning and biologically relevant models

The evolution of DILI toxicogenomics has brought forth deep learning, network-based learning, multimodal learning and graph-based learning. Given the methodological diversity of this phase, the studies are organized into two major paradigms: deep learning for transcriptomic prediction and mechanism-informed, network-based, and multimodal models.

#### 3.3.1 Deep learning for transcriptomic prediction

One advancement is illustrated by Feng et al. (2019). Using rat liver toxicogenomic expression profiles derived from the TG-GATEs/ArrayExpress databases, they developed a gene-expression-based deep neural network (DNN) model for DILI prediction. Their model outperformed an SVM comparator on a held-out evaluation set, achieving higher accuracy and area under the receiver operating characteristic curve (AUC). This study also underscores the importance of feature selection, as the best-performing reduced gene set was enriched for functions related to metabolism, liver and hepatobiliary development, and responses to toxic substances.

Chierici et al. (2020) presented a benchmark study using data from the CAMDA 2018 CMap Drug Safety Challenge. In this study, they compared the performance of deep learning algorithms relative to RF and DNN algorithms based on MCF7 and PC3 transcriptomic responses to 276 compounds. They found that classification performance for DILI status was poor with Matthews correlation coefficient (MCC) less than 0.2 for both cross-validation and external validation. This poor performance shows that transcriptomic AI models do not automatically generalize across datasets, cell lines, or DILI label definitions. In this case, the negative result suggests that algorithmic complexity alone cannot overcome weak biological signal, noisy endpoint annotation, limited sample size, or dataset-specific transcriptional artifacts which highlight the need for biologically structured features, better endpoint curation, and stronger external validation rather than simply more complex models.

Similarly, Li et al. (2020) utilized LINCS L1000 profiles along with annotations from DILIst dataset to create a DNN model that outperformed k-nearest neighbors (KNN), SVM, and RF. Meanwhile they also provided mechanistic interpretation for the selected significant genes using ingenuity pathway analysis (IPA), protein–protein interaction (PPI) network and GO interpretations.

Mohsen et al. (2021) utilized TG-GATEs toxicogenomic data with FDA Adverse Event Reporting System (FAERS) data for adverse drug reactions (ADRs) and performed a DNN-based framework for the prediction of multiple ADRs including liver-relevant ADRs like hepatitis fulminant and liver transplant. It integrates deep learning with Boruta feature selection, Optuna, and pathway enrichment, and identified a set of predictive genes related to xenobiotic metabolism NF-kappaB signaling complement and coagulation cascades as well as fatty acid degradation.

Together, these studies show that deep learning can improve DILI prediction, but only when the input data contains biologically meaningful signals. Poor benchmark performance also indicates that deeper models cannot overcome noisy labels, weak transcriptomic signal, or poor cross-dataset generalizability.

#### 3.3.2 Mechanism-informed, network-based, and multimodal models

AI is increasingly transforming toxicology by enabling the integration of toxicogenomic, imaging, and high-throughput screening data to uncover toxicity mechanisms that would be difficult to resolve through conventional analytical approaches (Lin and Chou, 2022). In this stage, the toxicogenomic studies are not only applied to compound classification but extend to understanding complex nonlinear relationships between biological endpoints (Valerio, 2025).

Jaume et al. (2024a) introduced a transcriptomics-guided multimodal pretraining framework (TANGLE) that aligned histology whole-slide images with gene expression profiles through contrastive learning. The study demonstrated improved few-shot lesion classification and highlighted the potential of molecularly informed representation learning for toxicologic pathology.

The pathway to this phase was significantly influenced by Kohonen et al. (2017), where a predictive toxicogenomics space (PTGS) was described based on transcriptomics and cytotoxicity data through probabilistic component modeling. They introduced a latent gene space representation comprising 14 overlapping components related to cytotoxicity that would be used for predicting dose-dependent cytotoxicity rat liver pathology in TG-GATEs and human DILI. The components were biologically interpretable.

Recently, Bergen et al. (2025) expanded the concept and built a large-scale human toxicogenomics database using RNA-seq data from primary human hepatocytes together with pharmacokinetic data to assess dose-resolved DILI risks. They utilized RF to develop ToxPredictor, which outperformed state-of-the-art methods on liver toxicity prediction and provides mechanistic insights into hepatotoxic pathways. Hellgren et al. (2026) developed a preclinical DILI risk screening tool, namely AEGIS (Apoptotic Effector Genes in Safety), to quantify dose-dependent gene expressions of apoptosis-regulating transcription factors from transcriptomic data. They demonstrated that these tools could be used for rapid screening of DILI risk at the early stage of drug discovery.

By integrating biological knowledge into network-based models, Shin et al. (2022) presented a semi-automated workflow that combined ToxCast, Comparative Toxicogenomics Database (CTD), Reactome, Open TG-GATEs, and GeneMANIA to build biological networks for cholestasis, steatosis, hepatitis, and cirrhosis diseases. The study developed disease-specific mechanistic networks and demonstrated the value of network-based modeling for DILI related phenotypes. Jin et al. (2024) introduced a mechanism-driven approach through entropy weighting together with eXtreme Gradient Boosting (XGBoost) and SHapley Additive exPlanations (SHAP) for assessing the relative importance of biological pathways in liver toxicity. Instead of relying only on individual genes, this study established an interpretable panel consisting of 11 pathways that spanned biological processes as well as stress responses.

These mechanism-informed, network-based, and multimodal models show a shift from prediction alone toward biological interpretation. Their strength lies in connecting transcriptomic data with pathways, networks, pharmacokinetics, histopathology, and disease-relevant biological context.

### 3.4 Generative and representation-learning AI for the study of DILI toxicogenomics

Recent advancements have highlighted the transformative potential of biologically informed, generative AI within toxicogenomics. Generative adversarial networks (GANs), in particular, have been increasingly deployed to synthesize omics data and model complex biological translations.

Early applications in this domain focused on data augmentation; for instance, Chen et al. (2022) developed ToxGAN to generate highly realistic synthetic toxicogenomic profiles. Building upon predictive toxicology, Chen et al. (2023) later introduced AnimalGAN, a model conditioned on chemical structure, drug class, and FDA approval year. Remarkably, AnimalGAN demonstrated hepatotoxicity assessment performance comparable to both traditional quantitative structure-activity relationship (QSAR) approaches and real *in vivo* animal data. ToxGAN and AnimalGAN are best interpreted primarily as data synthesis, augmentation, and phenotype simulation tools, rather than as direct mechanistic discovery models.

Furthermore, generative frameworks have proven instrumental in bridging spatial and experimental biological gaps. Addressing cross-organ prediction, Li et al. (2023) developed TransOrGAN to infer organ-specific toxicogenomic responses using data derived from alternate organs. This concept was subsequently advanced by Li et al. (2024) with TransTox, a framework enabling robust, bidirectional translation between liver and kidney transcriptomes. Most recently, Chandra et al. (2025) expanded these translational capabilities by introducing AIVIVE (AI-driven *in vitro* to *in vivo* extrapolation), a GAN-driven approach that intelligently anchors its predictions using biologically relevant gene modules. These studies form a second category of GAN-based models focused on cross-organ and in vitro–in vivo transcriptomic translation, rather than simple data augmentation. Their value lies in addressing translational gaps between organs, species, or experimental systems.

A third category includes representation-learning models for mechanism-oriented discovery. These models do not primarily synthesize new transcriptomic profiles or translate responses across organs, but instead learn biologically informed representations from molecular networks, toxicogenomic profiles, or histopathology images to support mechanistic interpretation. Xiao et al. (2025) provided a BioGL-GCN, a graph convolutional network (GCN)-based framework that integrates toxicogenomic profiles with PPI structure plus gene frequency information from enrichment analysis to improve DILI prediction. Jaume et al. (2024b) introduced a deep learning model called GEESE that predicts transcriptomic signals directly from hematoxylin and eosin (H&E)-stained rat liver whole slide images based on multiple instance learning using Vision Transformer, which produces pseudo-spatial gene expression maps that correlate with DILI-related lesions. This study establishes a quantitative relationship between morphology and transcriptomics which marks a further step toward multimodal mechanism-aware toxicology.

Collectively, these approaches show three distinct uses of advanced AI in DILI toxicogenomics: data synthesis and phenotype simulation, cross-organ or in vitro–in vivo transcriptomic translation, and representation learning for mechanism-oriented interpretation. Their mechanistic value is often indirect and depends on downstream pathway analysis, gene-module anchoring, multimodal validation, or biological validation.

## 4 Mechanistic insights from AI-driven DILI toxicogenomics

Several biological themes were identified from AI-driven toxicogenomic studies in literature (Table 2). Oxidative stress is the most common biological process found. De Abrew et al. (2015) reported enrichment pathways such as “response to oxidative stress” in clustering hepatotoxicants by their transcriptomic mode of action. Zhang L. et al. (2012) reported liver-blood signatures in acetaminophen (APAP)-induced liver injury that were closely related to mitochondrial and immune system disruption which are directly associated with oxidative stress. More recent AI-based studies extend these earlier observations by showing that oxidative stress pathway is not only enriched, but also repeatedly prioritized as a predictive and phenotype-relevant signal. The Shin et al. (2022) study established the connection between oxidative stress and downstream injury processes; Jin et al. (2024) identified NRF2-mediated oxidative stress response among 11 pathways by using an interpretable ML model XGBoost-SHAP. Zhao et al. (2024) showed that one cluster of predicted high-DILI-risk compounds is significantly associated with higher nuclear respiratory factor 1 (NRF1) and reactive oxygen species (ROS) signatures, as well as mitochondrial and endoplasmic reticulum dysfunction. This finding supports an oxidative-stress-linked mechanism in that subset of compounds.

**TABLE 2:** Recurrent mechanistic themes identified across DILI toxicogenomic studies.

Mechanistic theme	Representative studies	Example pathways/signals/representative biomarkers	AI/ML method used	Relevance to DILI interpretation
Oxidative stress	De Abrew et al. (2015), Zhang et al. (2012a), Shin et al. (2022), Jin et al. (2024), Zhao et al. (2024)	Response to oxidative stress; nuclear factor erythroid 2-related factor 2 (NRF2)-mediated oxidative stress response; oxidative injury-linked transcript clusters	Pathway enrichment; network analysis; XGBoost-SHAP; signature scoring	Recurrent upstream stress mechanism across multiple DILI phenotypes and model systems
Mitochondrial dysfunction	Zhang et al. (2012a), Shin et al. (2022), Zhao et al. (2024), Jaume et al. (2024b)	Mitochondrial function; oxidative phosphorylation; mitochondrial dysfunction; ER dysfunction; cellular stress/apoptosis	Co-expression analysis; network modeling; signature scoring; morphomolecular modeling	Mechanistic bridge between early toxic exposure, energy failure, and later injury progression
Xenobiotic metabolism/CYP perturbation	Rueda-Zárate et al. (2017), De Abrew et al. (2015), Kohonen et al. (2017), Feng et al. (2019), Mohsen et al. (2021), Zhao et al. (2024), Chandra et al. (2025)	CYP family 1 subfamily A; CYP inducer clusters; xenobiotic metabolism signaling; metabolism of xenobiotics by CYP; CYP recovery in IVIVE	GSEA; clustering; PTGS; DNN feature interpretation; IVIVE gene-module anchoring	Captures the interface between drug handling and hepatotoxic response, reinforcing the value of transcriptomics in DILI
Inflammation/immune signaling	Zhang et al. (2012a), Tawa et al. (2014), Mohsen et al. (2021), Shin et al. (2022)	Immune response; leukocyte activation; chemotaxis; leukocyte transendothelial migration; natural killer cell mediated cytotoxicity; NF-kappaB signaling	Co-expression modules; pathway enrichment; DNN feature selection; biological network modeling	Highlights the role of immune activation in injury amplification, remodeling, and phenotype diversification
Cell death/necrosis/apoptosis	Kohonen et al. (2017), Li et al. (2020), Zhao et al. (2024), Jaume et al. (2024b), Xiao et al. (2025)	Liver necrosis/cell death; GADD45 signaling; ATM signaling; apoptotic process; necrosis-associated lesions; p53/MAPK/DNA-damage pathways	PTGS; pathway/PPI analysis; lesion classification; GCN/PPI modeling	Defines a core injury-response program repeatedly recovered across AI-enabled toxicogenomic models
Cholestatic pathways	Kohonen et al. (2017), Jiang et al. (2023), Shin et al. (2022), Zhao et al. (2024)	Hepatic cholestasis; bile acid synthesis; bile flow disruption; cholestasis disease networks	ML feature selection; pathway enrichment; disease network modeling	Supports subtype-specific interpretation beyond broad toxic vs. non-toxic classification
Steatotic/lipid dysregulation pathways	Tawa et al. (2014), Aguayo-Orozco et al. (2021), Shin et al. (2022)	Steatosis signatures; periportal lipid accumulation; bile secretion; NAFLD-related pathways	Co-expression modules; pathway fingerprints; biological network modeling	Indicates that transcriptomic AI can recover lipid-handling disturbances linked to specific injury phenotypes
Fibrotic/tissue remodeling pathways	Tawa et al. (2014), Kohonen et al. (2017), Shin et al. (2022)	Hepatic fibrosis; phagosome; regulation of actin cytoskeleton; fibrosis-related immune remodeling; hepatic stellate cell activation	Co-expression modules; PTGS; disease network modeling	Shows progression from acute injury signals toward chronic remodeling and fibrosis-relevant biology

Another top-ranking pathway is mitochondrial dysfunction, which often accompanies oxidative stress. Zhang L. et al. (2012) found the shared liver-blood signature after 6-h drug treatments were strongly related to mitochondrial function and oxidative phosphorylation; at 24-h, the genes related to mitochondria were still part of the reduced shared signature tracking the status of liver injury. Shin et al. (2022) employed biologically informed approaches to study the biological network across multiple phenotypes such as cholestasis, steatosis, hepatitis, and cirrhosis. They found all these phenotypes are closely related to mitochondrial dysfunction. Zhao et al. (2024) found an association between high-risk compound-induced liver injury with mitochondrial and endoplasmic reticulum dysfunction concerning pathways. Collectively, these findings support mitochondrial dysfunction as a key mechanism linking transcriptomic injury markers. The added value of AI is that it helps connect mitochondrial signals with predictive outputs, specific injury phenotypes, and high-risk compound groups rather than treating mitochondrial enrichment as an isolated pathway result.

The third common mechanism is altered xenobiotic metabolism and cytochrome P450 (CYP) dysfunction. In the work of Rueda-Zárate et al. (2017), cytochrome P450 family one subfamily A was noted in the pathway for xenobiotic metabolism. Furthermore, De Abrew et al. (2015) found some clusters related to cytochrome P450 inducers and drug responses. Xenobiotic metabolism is also the key mechanism in Kohonen et al. (2017) and Mohsen et al. (2021) studies, as is the successful translation from *in vitro* to *in vivo* related to CYP expression in Chandra et al. (2025) study. These examples show that AI-driven models can help determine when xenobiotic metabolism signals are not only biologically plausible, but also useful for prediction, cross-system comparison, and translational modeling.

The pathways of inflammation, immune response and cell death were reported. Zhang L. et al. (2012) identified immune response and activation of leukocytes with chemotaxis in APAP-associated liver injury. Modules related to fibrosis were reported by Tawa et al. (2014) which had a connection to leukocyte migration and immune cytotoxicity. Injury-related modules were reported connecting with necrosis, apoptosis, stress response, and morphology related to lesions by Kohonen et al. (2017), Zhao et al. (2024), and Jaume et al. (2024b). These studies show a shift from pathway listing toward phenotype-linked interpretation, connecting molecular signatures with pathology, morphology, and lesion-specific patterns.

**FIGURE 3:**
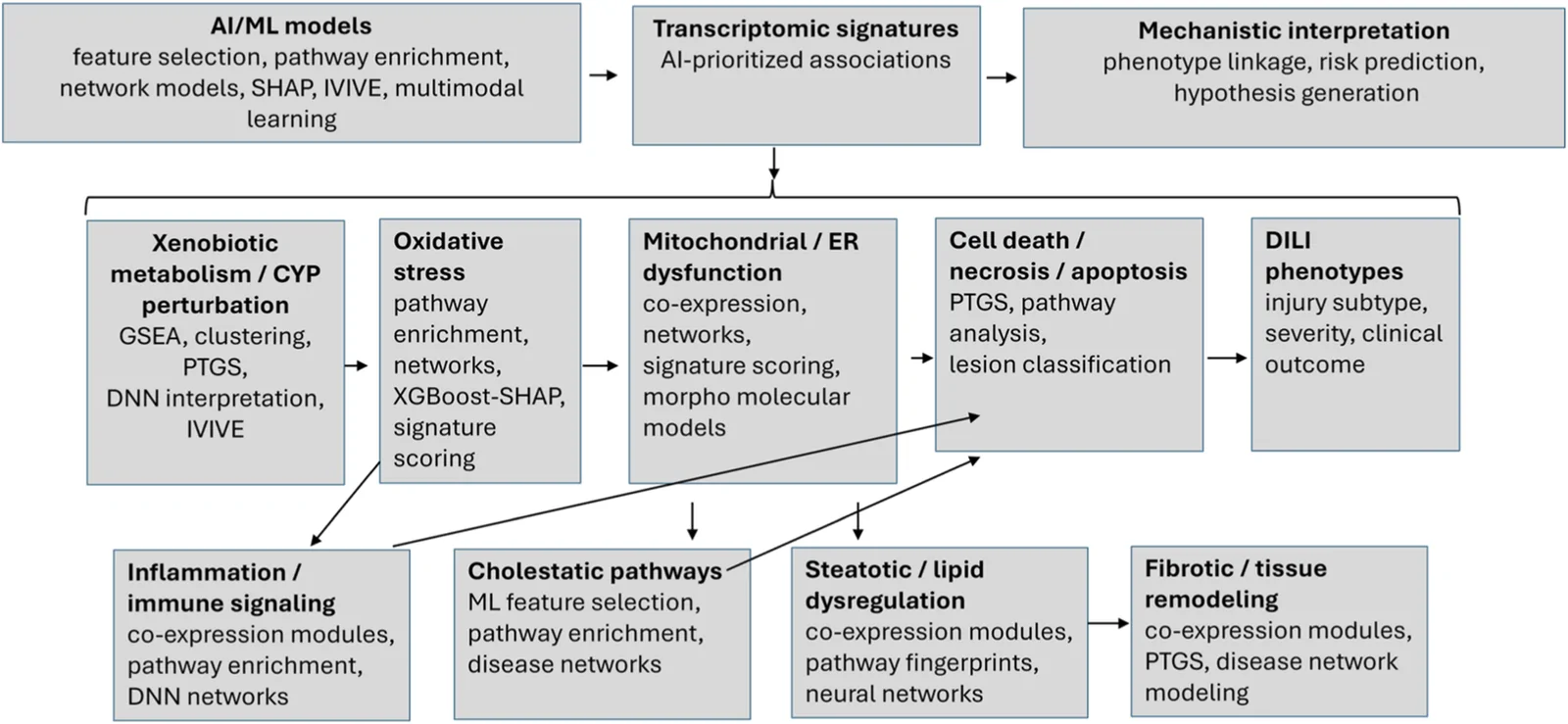
AI-prioritized mechanistic pathways and interconnections in DILI toxicogenomics. The figure summarizes how AI/ML models prioritize transcriptomic signatures associated with major candidate DILI mechanisms, including xenobiotic metabolism/CYP perturbation, oxidative stress, mitochondrial and ER dysfunction, inflammation, cholestatic pathways, steatotic/lipid dysregulation, cell death, fibrotic remodeling, and DILI phenotypes. The arrows indicate commonly reported biological relationships among AI-prioritized transcriptomic signatures. These pathways should be interpreted as candidate mechanistic associations rather than confirmed causal mechanisms unless functionally validated.

Overall, AI-driven DILI toxicogenomics strengthens mechanistic interpretation by prioritizing predictive pathways and linking transcriptomic signatures with injury phenotypes (Figure 3), but these associations remain hypothesis-generating unless functionally validated. While AI-driven models have improved DILI prediction, many identified mechanisms, including oxidative stress, mitochondrial dysfunction, xenobiotic metabolism, inflammation, and cell death, were already established in earlier DILI biology. The main contribution of AI is therefore not the discovery of entirely new mechanisms, but the prioritization, integration, and translation of these mechanisms across high-dimensional datasets. AI-derived transcriptomic signatures should be interpreted as candidate mechanistic associations rather than causal mechanisms unless supported by functional validation. Because transcriptomic data capture changes in gene expression rather than direct perturbation of biological pathways, transcriptomics alone cannot establish causal mechanisms without functional validation using experimental, biochemical, or perturbational approaches.

## 5 Current limitations in translational modeling

A primary limitation remains the biological discordance between standard *in vitro* assays and complex *in vivo* human liver responses. While generative frameworks like AIVIVE (discussed in Section 3.4) represent pioneering computational attempts to bridge this gap, widespread clinical translation is still hindered by issues such as poor annotations, insufficient external validation, and limited integration of relevant clinical biological information.

One limitation lies in translational modeling between *in vitro* transcriptional data and *in vivo* liver biology. Sutherland et al. (2016) demonstrated that although concordance within rat liver datasets was significant, much lower concordance was observed when transcriptional data was compared between liver and cultured hepatocyte systems. This is not just a technical limitation of data analysis; it can be shown by the fact that discordance exists at gene, gene-set, and WGCNA levels. Cultured hepatocytes had a substantial baseline shift characterized by loss of differentiation, reduced biological processes such as fatty acid oxidation and CYP metabolism.

Another issue is that increased model complexity does not always compensate for poorly informative data. Comparative benchmarking of multiple ML algorithms for DILI prediction demonstrated that Bayesian, SVM, KNN, AdaBoost, and deep learning models achieved broadly similar predictive performance, suggesting that dataset quality and endpoint definition may remain stronger determinants of model success than algorithmic complexity alone (Minerali et al., 2020). Chierici et al. (2020) showed that despite testing several machine learning methods including random forests and multilayer perceptron deep learning networks, clinical DILI data was not improved. Different train and test partitions did not solve the issue.

A further challenge arises from ambiguous endpoint definitions and inconsistent benchmarking. A major barrier to robust AI-based DILI prediction remains the limited size, and inconsistent annotation of available DILI datasets, which restrict fair benchmarking and generalizability of predictive models (Vall et al., 2021). Liu et al. (2020) demonstrated that prediction was substantially complicated by the poor definition of DILI risk, and the selection of negative samples.

One major limitation is the lack of prospective clinical validation for transcriptomic DILI biomarkers. Although several AI/ML models report promising retrospective performance, most were developed using curated public datasets, preclinical systems, or benchmark labels. Their ability to guide prospective drug development decisions, clinical trial monitoring, or patient-level DILI risk prediction remains largely unproven.

Species-specific transcriptional differences also limit animal-to-human translation. Rat liver, primary human hepatocytes, HepG2 cells, and other *in vitro* systems differ in baseline gene expression, metabolic competence, CYP activity, immune context, and injury progression. Therefore, even strong performance in one biological system may not translate directly to human DILI prediction.

Relatedly, dataset heterogeneity caused by batch effects, platform differences, and limited positive DILI cases remains a major barrier to model generalizability (Luo et al., 2010). Reviewed studies use microarray, RNA-seq, LINCS L1000/CMap, TG-GATEs, DrugMatrix, GEO, and other sources with different preprocessing methods, dose levels, exposure times, tissue types, and biological systems. These differences can cause models to learn platform- or dataset-specific signals rather than generalizable DILI biology. In addition, severe DILI-positive cases are relatively limited, which contributes to class imbalance, unstable feature selection, and inflated performance estimates in small training datasets.

Regulatory acceptance is another key challenge for AI-based safety tools (Meier et al., 2025). For such models to support drug development decisions, they will require transparent endpoint definitions, reproducible preprocessing, clearly defined applicability domains, external and preferably prospective validation, uncertainty estimation, and evidence that predictions improve safety assessment beyond conventional assays.

This review focuses on bulk and perturbational transcriptomic AI/ML studies; however, future work should incorporate single-cell and spatial approaches to better resolve cell-type-specific hepatocyte, cholangiocyte, immune-cell, stellate-cell, and endothelial responses that may be obscured in bulk profiles.

## 6 Future directions

The future of DILI toxicogenomics will likely be multimodal, more clinically relevant, biologically contextualized, and integrative, with an emphasis on developing dynamic models capable of capturing complex phenotypic states. For example, Quintás et al. (2021) showed that serum metabolomics can be more representative for dynamic states of liver toxicity like cholestatic, hepatocellular, mixed, and recovery.

Proteomics can also complement transcriptomic AI models by providing clinically measurable DILI biomarkers. Ravindra et al. (2023) identified serum protein candidates such as ACO1, ASS1, FAH, FBP1, and CPS1 that separated DILI onset from healthy controls with high AUC values, while FBP1 alone or combined with GSTA1 and LECT2 showed potential for distinguishing DILI from acute non-DILI liver injury.

Another direction could integrate experimental context into model training (Wang et al., 2022). Masood et al. (2025) showed that pre-training with large-scale information related to *in vitro* assays could improve pre-clinical DILI prediction. Recent work has demonstrated that transformer-based AI models integrating non-destructive human liver organoid brightfield imaging can achieve clinically relevant ternary DILI severity classification, highlighting the translational promise of multimodal, biologically realistic toxicity prediction frameworks (Tan et al., 2025).

Recent DILI-specific work has shown that integrating rat liver transcriptomics with quantum mechanical descriptors and physiologically based rat-to-human exposure metrics can improve AI/ML prediction in data-limited settings, supporting the value of knowledge-enriched multimodal modeling for DILI risk assessment (Bhave et al., 2025).

More broadly, although toxicogenomics has remained the central mechanistic framework, other layers, such as microbiome-informed, histopathologic, and assay-informed data are continuously added (Meier et al., 2025). More recently, an industry-wide Consortium, namely Omics for Assessing Signatures for Integrated Safety (OASIS), was established by Rouquié et al. (2025), aiming to combine multi-omics technologies (including transcriptomics, proteomics, and Cell Painting) and *in vitro* model to transform chemical safety assessment. Explainable AI in DILI toxicogenomics could extend beyond SHAP-based feature attribution toward broader interpretable machine learning strategies, including intrinsically interpretable models guided by toxicological knowledge frameworks and *post hoc* approaches such as perturbation-based and approximation-based explanations (Jia et al., 2023). DILI-specific XAI work has also moved beyond SHAP-based transcriptomic interpretation. For example, GeoDILI used a geometry-enhanced graph neural network to predict DILI and derive interpretable molecular structural alerts, showing how graph-based explanations can support safer compound prioritization and mechanistic hypothesis generation (Wu et al., 2023).

Beyond GAN-based models, future DILI research could also evaluate other generative architectures, including diffusion models, which have shown strong potential for 3D molecular generation, target-conditioned design, docking, conformer generation, and fragment-based drug design, but remain largely unexplored in DILI-specific toxicogenomic benchmarks (Alakhdar et al., 2024).

Data availability has been a major strength of DILI toxicogenomics, with many reviewed studies relying on public resources such as TG-GATEs, DrugMatrix, LINCS L1000/CMap, GEO, FAERS, CTD, PubChem, DrugBank, and DILIst annotations. However, the availability of processed datasets, code, trained models, benchmark labels, and train-test splits remains inconsistent across studies. Future work should prioritize sharing raw and processed omics data, compound identifiers, dose/time metadata, endpoint definitions, preprocessing workflows, model code, trained weights, and external validation results to improve reproducibility and fair benchmarking.

## 7 Conclusion

AI and ML in DILI toxicogenomics research have advanced significantly in the past decade. These developments collectively demonstrate that toxicogenomics is not just another high-dimensional input space. The field has transcended its initial focus on rudimentary pattern recognition for early hepatotoxicity detection; instead, it has emerged as a central molecular framework capable of capturing complex, critical mechanisms of toxicity, such as oxidative stress, mitochondrial injury, altered xenobiotic metabolism, and phenotype-specific injury pathways. While methodological sophistication has outpaced clinical integration, several challenges continue to impede bedside translation. Specifically, in vitro-in vivo (IVIVE) discordance, heterogeneous endpoints, limited comprehensive validation, and incomplete biological context remain significant bottlenecks. Although other technologies, such as metabolomics, microbiome-informed research, computational pathology, and assay-informed modeling hold substantial translational promise, the most exciting frontier may still remain the application of AI in toxicogenomics to achieve deeper, mechanistically anchored insights.

## References

[B1] Afshari C. A. Nuwaysir E. F. Barrett J. C. (1999). Application of complementary DNA microarray technology to carcinogen identification, toxicology, and drug safety evaluation. Cancer Res. 59 (19), 4759–4760. 10519378

[B2] Aguayo-Orozco A. Brunak S. Taboureau O. (2021). Extrapolation of drug induced liver injury responses from cancer cell lines using machine learning approaches. Comput. Toxicol. 17, 100147. 10.1016/j.comtox.2020.100147

[B3] Aguirre-Plans J. Piñero J. Souza T. Callegaro G. Kunnen S. J. Sanz F. (2021). An ensemble learning approach for modeling the systems biology of drug-induced injury. Biol. Direct 16, 5. 10.1186/s13062-020-00288-x 33435983 PMC7805064

[B4] Alakhdar A. Poczos B. Washburn N. (2024). Diffusion Models in *de novo* Drug Design. J. Chem. Inf. Model. 64, 7238–7256. 10.1021/acs.jcim.4c01107 39322943 PMC11481093

[B5] Andrade R. J. Chalasani N. Björnsson E. S. Suzuki A. Kullak-Ublick G. A. Watkins P. B. (2019). Drug-induced liver injury. Nat. Rev. Dis. Prim. 5, 58. 10.1038/s41572-019-0105-0 31439850

[B6] Bergen V. Kodella K. Srikrishnan S. Barrandon O. Anderson S. Rogers-Grazado M. (2025). A large-scale human toxicogenomics resource for drug-induced liver injury prediction. Nat. Commun. 16, 9860. 10.1038/s41467-025-65690-3 41233320 PMC12615732

[B7] Bhave R. Bello B. Bhatt D. Machulcz J. Shea J. A. R. Khotimchenko M. (2025). AI-Driven integration of transcriptomics, quantum mechanics, and physiology for predicting drug-induced liver injury in data-limited scenarios. Chem. Res. Toxicol. 38, 1325–1335. 10.1021/acs.chemrestox.4c00542 40598914

[B8] Chalasani N. Hayashi P. H. Luffer-Atlas D. Regev A. Watkins P. B. (2025). Assessment of liver injury potential of investigational medicines in drug development. Hepatology. 10.1097/HEP.0000000000001281 39999469

[B9] Chandra M. Li T. Tong W. (2025). AIVIVE: a novel AI framework for enhanced *in vitro* to *in vivo* extrapolation (IVIVE) of toxicogenomics data. Toxicol. Sci. 207, 361–371. 10.1093/toxsci/kfaf100 40692113 PMC12469192

[B10] Chen M. Vijay V. Shi Q. Liu Z. Fang H. Tong W. (2011). FDA-approved drug labeling for the study of drug-induced liver injury. Drug Discov. Today 16, 697–703. 10.1016/j.drudis.2011.05.007 21624500

[B11] Chen M. Zhang M. Borlak J. Tong W. (2012). A decade of toxicogenomic research and its contribution to toxicological science. Toxicol. Sci. 130, 217–228. 10.1093/toxsci/kfs223 22790972

[B12] Chen M. Borlak J. Tong W. (2014). Predicting idiosyncratic drug-induced liver injury – some recent advances. Expert Rev. Gastroenterol. Hepatol. 8, 721–723. 10.1586/17474124.2014.922871 24857265

[B13] Chen X. Roberts R. Tong W. Liu Z. (2022). Tox-GAN: an artificial intelligence approach alternative to animal Studies—A case Study with toxicogenomics. Toxicol. Sci. 186, 242–259. 10.1093/toxsci/kfab157 34971401

[B14] Chen X. Roberts R. Liu Z. Tong W. (2023). A generative adversarial network model alternative to animal studies for clinical pathology assessment. Nat. Commun. 14, 7141. 10.1038/s41467-023-42933-9 37932302 PMC10628291

[B15] Chierici M. Francescatto M. Bussola N. Jurman G. Furlanello C. (2020). Predictability of drug-induced liver injury by machine learning. Biol. Direct 15, 3. 10.1186/s13062-020-0259-4 32054490 PMC7020573

[B16] De Abrew K. N. Overmann G. J. Adams R. L. Tiesman J. P. Dunavent J. Shan Y. K. (2015). A novel transcriptomics based *in vitro* method to compare and predict hepatotoxicity based on mode of action. Toxicology 328, 29–39. 10.1016/j.tox.2014.11.008 25475144

[B17] Feng C. Chen H. Yuan X. Sun M. Chu K. Liu H. (2019). Gene expression data based deep learning model for accurate prediction of drug-induced liver injury in advance. J. Chem. Inf. Model. 59, 3240–3250. 10.1021/acs.jcim.9b00143 31188585

[B18] Ganter B. Tugendreich S. Pearson C. I. Ayanoglu E. Baumhueter S. Bostian K. A. (2005). Development of a large-scale chemogenomics database to improve drug candidate selection and to understand mechanisms of chemical toxicity and action. J. Biotechnol. 119, 219–244. 10.1016/j.jbiotec.2005.03.022 16005536

[B19] García-Cañaveras J. C. Castell J. V. Donato M. T. Lahoz A. (2016). A metabolomics cell-based approach for anticipating and investigating drug-induced liver injury. Sci. Rep. 6, 27239. 10.1038/srep27239 27265840 PMC4893700

[B20] Hellgren J. Chouhan B. Uatay A. Elgendy R. Lindgren J. Toki N. (2026). Apoptotic signatures allow early and rapid screening of drug-induced liver injury to accelerate drug discovery. Commun. Med. 6, 48. 10.1038/s43856-025-01306-7 41444459 PMC12827481

[B21] Jaume G. Oldenburg L. Vaidya A. Chen R. J. Williamson D. F. K. Peeters T. (2024a). “Transcriptomics-Guided slide representation learning in computational pathology,” in 2024 IEEE/CVF Conf. Comput. Vis. Pattern Recognit. (CVPR) (Seattle, WA, USA: IEEE), 9632–9644. 10.1109/CVPR52733.2024.00920

[B22] Jaume G. Peeters T. Song A. H. Pettit R. Williamson D. F. K. Oldenburg L. (2024b). AI-driven discovery of morphomolecular signatures in toxicology. bioRxiv. 10.1101/2024.07.19.604355 39091765 PMC11291055

[B23] Jia X. Wang T. Zhu H. (2023). Advancing computational toxicology by interpretable machine learning. Environ. Sci. Technol. 57, 17690–17706. 10.1021/acs.est.3c00653 37224004 PMC10666545

[B24] Jiang J. Van Ertvelde J. Ertaylan G. Peeters R. Jennen D. De Kok T. M. (2023). Unraveling the mechanisms underlying drug-induced cholestatic liver injury: identifying key genes using machine learning techniques on human *in vitro* data sets. Arch. Toxicol. 97, 2969–2981. 10.1007/s00204-023-03583-4 37603094 PMC10504391

[B25] Jin Y. Shou Y. Lei Q. Du C. Xu L. Chen N. (2024). An entropy weight method to integrate big omics and mechanistically evaluate DILI. Hepatology 79, 1264–1278. 10.1097/HEP.0000000000000628 37820269 PMC11095888

[B26] Kohonen P. Parkkinen J. A. Willighagen E. L. Ceder R. Wennerberg K. Kaski S. (2017). A transcriptomics data-driven gene space accurately predicts liver cytopathology and drug-induced liver injury. Nat. Commun. 8, 15932. 10.1038/ncomms15932 28671182 PMC5500850

[B27] Li T. Tong W. Roberts R. Liu Z. Thakkar S. (2020). Deep learning on high-throughput transcriptomics to predict drug-induced liver injury. Front. Bioeng. Biotechnol. 8, 562677. 10.3389/fbioe.2020.562677 33330410 PMC7728858

[B28] Li T. Roberts R. Liu Z. Tong W. (2023). TransOrGAN: an artificial intelligence mapping of rat transcriptomic profiles between organs, ages, and sexes. Chem. Res. Toxicol. 36, 916–925. 10.1021/acs.chemrestox.3c00037 37200521 PMC10433534

[B29] Li T. Chen X. Tong W. (2024). Bridging organ transcriptomics for advancing multiple organ toxicity assessment with a generative AI approach. Npj Digit. Med. 7, 310. 10.1038/s41746-024-01317-z 39501092 PMC11538515

[B30] Lin Z. Chou W.-C. (2022). Machine learning and artificial intelligence in toxicological sciences. Toxicol. Sci. 189, 7–19. 10.1093/toxsci/kfac075 35861448 PMC9609874

[B31] Liu Y. Ai N. Keys A. Fan X. Chen M. (2015). Network pharmacology for traditional Chinese medicine research: methodologies and applications. Chin. Herb. Med. 7, 18–26. 10.1016/S1674-6384(15)60015-6

[B32] Liu Z. Huang R. Roberts R. Tong W. (2019). Toxicogenomics: a 2020 vision. Trends Pharmacol. Sci. 40, 92–103. 10.1016/j.tips.2018.12.001 30594306 PMC9988209

[B33] Liu X. Zheng D. Zhong Y. Xia Z. Luo H. Weng Z. (2020). Machine‐Learning prediction of oral drug‐induced liver injury (DILI) *via* multiple features and endpoints. Biomed. Res. Int. 2020, 4795140. 10.1155/2020/4795140 32509859 PMC7254069

[B34] Luo J. Schumacher M. Scherer A. Sanoudou D. Megherbi D. Davison T. (2010). A comparison of batch effect removal methods for enhancement of prediction performance using MAQC-II microarray gene expression data. Pharmacogenomics J. 10, 278–291. 10.1038/tpj.2010.57 20676067 PMC2920074

[B35] Masood M. A. Nagaraja A. A. Belaid K. Mesens N. Ceulemans H. Kaski S. (2025). VitroBert: modeling DILI by pretraining BERT on *in vitro* data. J. Cheminform. 17, 119. 10.1186/s13321-025-01048-7 40770816 PMC12326598

[B36] Meier M. J. Harrill J. Johnson K. Thomas R. S. Tong W. Rager J. E. (2025). Progress in toxicogenomics to protect human health. Nat. Rev. Genet. 26, 105–122. 10.1038/s41576-024-00767-1 39223311

[B37] Minerali E. Foil D. H. Zorn K. M. Lane T. R. Ekins S. (2020). Comparing machine learning algorithms for predicting drug-induced liver injury (DILI). Mol. Pharm. 17, 2628–2637. 10.1021/acs.molpharmaceut.0c00326 32422053 PMC7702310

[B38] Mohsen A. Tripathi L. P. Mizuguchi K. (2021). Deep learning prediction of adverse drug reactions in drug discovery using open TG–GATEs and FAERS databases. Front. Drug Discov. 1, 768792. 10.3389/fddsv.2021.768792

[B39] Moreno-Torres M. Quintás G. Martínez-Sena T. Jover R. Castell J. V. (2024). Exploring individual variability in drug-induced liver injury (DILI) responses through metabolomic analysis. Int. J. Mol. Sci. 25, 3003. 10.3390/ijms25053003 38474249 PMC10932304

[B40] Quintás G. Martínez-Sena T. Conde I. Pareja Ibars E. Kleinjans J. Castell J. V. (2021). Metabolomic analysis to discriminate drug-induced liver injury (DILI) phenotypes. Arch. Toxicol. 95, 3049–3062. 10.1007/s00204-021-03114-z 34274980 PMC8380240

[B41] Ravindra K. C. Vaidya V. S. Wang Z. Federspiel J. D. Virgen-Slane R. Everley R. A. (2023). Tandem mass tag-based quantitative proteomic profiling identifies candidate serum biomarkers of drug-induced liver injury in humans. Nat. Commun. 14, 1215. 10.1038/s41467-023-36858-6 36869085 PMC9984368

[B42] Roth A. Boess F. Landes C. Steiner G. Freichel C. Plancher J. (2011). Gene expression‐based *in vivo* and *in vitro* prediction of liver toxicity allows compound selection at an early stage of drug development. J. Biochem. Mol. Toxicol. 25, 183–194. 10.1002/jbt.20375 21671310

[B43] Rouquié D. Bender A. Cheah J. Crute C. E. Dalmas D. Ewald J. (2025). The OASIS Consortium: integrating multi-omics technologies to transform chemical safety assessment. Toxicol. Sci. 208, 225–232. 10.1093/toxsci/kfaf128 40971841 PMC12646590

[B44] Rueda-Zárate H. A. Imaz-Rosshandler I. Cárdenas-Ovando R. A. Castillo-Fernández J. E. Noguez-Monroy J. Rangel-Escareño C. (2017). A computational toxicogenomics approach identifies a list of highly hepatotoxic compounds from a large microarray database. PLoS ONE 12, e0176284. 10.1371/journal.pone.0176284 28448553 PMC5407788

[B45] Ruepp S. Boess F. Suter L. Devera M. Steiner G. Steele T. (2005). Assessment of hepatotoxic liabilities by transcript profiling. Toxicol. Appl. Pharmacol. 207, 161–170. 10.1016/j.taap.2005.05.008 15990132

[B46] Shin H. K. Florean O. Hardy B. Doktorova T. Kang M.-G. (2022). Semi-automated approach for generation of biological networks on drug-induced cholestasis, steatosis, hepatitis, and cirrhosis. Toxicol. Res. 38, 393–407. 10.1007/s43188-022-00124-6 35865277 PMC9247124

[B47] Shin S. Lee C. Park T. (2025). Comprehensive analysis of high-throughput transcriptomics to distinguish drug-induced liver injury (DILI) phenotypes. Arch. Toxicol. 99, 3721–3734. 10.1007/s00204-025-04089-x 40464974 PMC12408718

[B48] Shrimali S. Chen M. Li D. Tong W. (2025). New approach methodologies (NAMs) for drug-induced liver injury (DILI): where are we now? Drug Discov. Today 30, 104452. 10.1016/j.drudis.2025.104452 40803573

[B49] Smith B. P. Auvil L. S. Welge M. Bushell C. B. Bhargava R. Elango N. (2020). Identification of early liver toxicity gene biomarkers using comparative supervised machine learning. Sci. Rep. 10, 19128. 10.1038/s41598-020-76129-8 33154507 PMC7645727

[B50] Sutherland J. J. Jolly R. A. Goldstein K. M. Stevens J. L. (2016). Assessing concordance of drug-induced transcriptional response in rodent liver and cultured hepatocytes. PLoS Comput. Biol. 12, e1004847. 10.1371/journal.pcbi.1004847 27028627 PMC4814051

[B51] Suzuki A. Chen M. (2025). Epidemiology and risk determinants of drug‐induced liver injury: current knowledge and future research needs. Liver Int. 45, e16146. 10.1111/liv.16146 39494620

[B52] Tan S. Ding Y. Wang W. Rao J. Cheng F. Zhang Q. (2025). Development of an AI model for DILI-level prediction using liver organoid brightfield images. Commun. Biol. 8, 886. 10.1038/s42003-025-08205-6 40483291 PMC12145446

[B53] Tawa G. J. AbdulHameed M. D. M. Yu X. Kumar K. Ippolito D. L. Lewis J. A. (2014). Characterization of chemically induced liver injuries using gene Co-Expression modules. PLoS ONE 9, e107230. 10.1371/journal.pone.0107230 25226513 PMC4165895

[B54] Uehara T. Ono A. Maruyama T. Kato I. Yamada H. Ohno Y. (2010). The Japanese toxicogenomics project: application of toxicogenomics. Mol. Nutr. Food Res. 54, 218–227. 10.1002/mnfr.200900169 20041446

[B55] Valerio L. G. (2025). Toxicological mechanisms: moving forward with artificial intelligence. Toxicol. Mech. Methods 35, 1139–1140. 10.1080/15376516.2025.2540458 40717362

[B56] Vall A. Sabnis Y. Shi J. Class R. Hochreiter S. Klambauer G. (2021). The promise of AI for DILI prediction. Front. Artif. Intell. 4, 638410. 10.3389/frai.2021.638410 33937745 PMC8080874

[B57] Wang M.-G. Wu S.-Q. Zhang M.-M. He J.-Q. (2022). Urine metabolomics and microbiome analyses reveal the mechanism of anti-tuberculosis drug-induced liver injury, as assessed for causality using the updated RUCAM: a prospective study. Front. Immunol. 13, 1002126. 10.3389/fimmu.2022.1002126 36483548 PMC9724621

[B58] Wu W. Qian J. Liang C. Yang J. Ge G. Zhou Q. (2023). GeoDILI: a robust and interpretable model for drug-induced liver injury prediction using graph neural network-based molecular geometric representation. Chem. Res. Toxicol. 36, 1717–1730. 10.1021/acs.chemrestox.3c00199 37839069

[B59] Xiao T. Liu Y. Hu K. Guo K. Zhang M. Wang T. (2025). Drug-induced liver injury prediction based on graph convolutional networks and toxicogenomics. PLoS Comput. Biol. 21, e1013423. 10.1371/journal.pcbi.1013423 40911636 PMC12412982

[B60] Yang Y. Blomme E. A. G. Waring J. F. (2004). Toxicogenomics in drug discovery: from preclinical studies to clinical trials. Chem. Biol. Interact. 150, 71–85. 10.1016/j.cbi.2004.09.013 15522262

[B61] Zhang L. Bushel P. R. Chou J. Zhou T. Watkins P. B. (2012a). Identification of identical transcript changes in liver and whole blood during Acetaminophen toxicity. Front. Genet. 3, 162. 10.3389/fgene.2012.00162 22973295 PMC3432993

[B62] Zhang M. Chen M. Tong W. (2012b). Is toxicogenomics a more reliable and sensitive biomarker than conventional indicators from rats to predict drug-induced liver injury in humans? Chem. Res. Toxicol. 25, 122–129. 10.1021/tx200320e 22122743

[B63] Zhao Y. Park J. Y. Yang D. Zhang M. (2024). A computational framework to *in silico* screen for drug-induced hepatocellular toxicity. Toxicol. Sci. 201, 14–25. 10.1093/toxsci/kfae078 38902949 PMC11347774

